# Lipopolysaccharide induced inflammation in the perivascular space in lungs

**DOI:** 10.1186/1745-6673-3-17

**Published:** 2008-07-30

**Authors:** Thomas Tschernig, Kyathanahalli S Janardhan, Reinhard Pabst, Baljit Singh

**Affiliations:** 1 Dept. of Functional and Applied Anatomy -4120-, Medical School of Hannover, Carl-Neuberg-Str. 1, 30625, Hannover, Germany; 2 Immunology Research Group, Departments of Veterinary Biomedical Sciences and Veterinary Microbiology, Western College of Veterinary Medicine, University of Saskatchewan, 52 Campus Drive, Saskatoon, SK, S7N 5B4, Canada; 3 Diagnostic Medicine and Pathobiology, 1800 Denison Avenue, Kansas State University, Manhattan, Kansas 66506, USA

## Abstract

**Background:**

Lipopolysaccharide (LPS) contained in tobacco smoke and a variety of environmental and occupational dusts is a toxic agent causing lung inflammation characterized by migration of neutrophils and monocytes into alveoli. Although migration of inflammatory cells into alveoli of LPS-treated rats is well characterized, the dynamics of their accumulation in the perivascular space (PVS) leading to a perivascular inflammation (PVI) of pulmonary arteries is not well described.

**Methods:**

Therefore, we investigated migration of neutrophils and monocytes into PVS in lungs of male Sprague-Dawley rats treated intratracheally with *E. coli *LPS and euthanized after 1, 6, 12, 24 and 36 hours. Control rats were treated with endotoxin-free saline. H&E stained slides were made and immunohistochemistry was performed using a monocyte marker and the chemokine Monocyte-Chemoattractant-Protein-1 (MCP-1). Computer-assisted microscopy was performed to count infiltrating cells.

**Results:**

Surprisingly, the periarterial infiltration was not a constant finding in each animal although LPS-induced alveolitis was present. A clear tendency was observed that neutrophils were appearing in the PVS first within 6 hours after LPS application and were decreasing at later time points. In contrast, mononuclear cell infiltration was observed after 24 hours. In addition, MCP-1 expression was present in perivascular capillaries, arteries and the epithelium.

**Conclusion:**

PVI might be a certain lung reaction pattern in the defense to infectious attacks.

## Background

Lipopolysaccharide (LPS) is a glycolipid of gram-negative bacterial cell walls and is present in many different airborne particles, such as tobacco smoke and a variety of environmental and occupational dusts [1,2]. Inhalation of LPS in man and administration through various routes in animal models result in inflammation [3,4]. LPS induces inflammatory cell signalling through its binding to LPS binding protein and subsequent interaction with Toll like receptor-4 (TLR-4) and other molecules such as CD14 and MD2 [5-7]. Lungs from LPS-treated animals show recruitment of neutrophils and monocytes into alveolar and vascular compartments through a complex interplay of cytokines, chemokines and adhesion molecules [8].

Recently, we identified the perivascular space (PVS) around pulmonary arteries as a unique morphological compartment with possible impact on inflammatory responses in the lung [9,10]. The PVS of pulmonary arteries increases in size in inflammation due to influx of fluids and inflammatory cells, which may come from perivascular capillaries that follow the arteries [11]. In addition, lymph vessels are located in the PVS and run in the opposite direction to the central draining lymph nodes. While the PVS is increased in various models of lung inflammation, anti-inflammatory agents such as anti-IL-9 agent reduce the amount of cellular infiltrates within this area [10,12]. It appears that the PVS may be an important location for the accumulation and actions of inflammatory cells in acute and chronic lung inflammation. Theogaraj et al. [13] found the PVS of the rat rich in white cells, including T- and B-lymphocytes, and suggested a significant role in host defence for this compartment.

Currently, there are no data on the temporary migration of inflammatory cells into the PVS in LPS-induced lung inflammation. Therefore, we conducted this study to define the kinetics of neutrophils and monocytes/macrophages into PVS in lungs of LPS-treated rats. In addition, we examined the immune-histological expression of monocyte chemoattractant protein-1 (MCP-1) in PVS of normal and LPS-treated rats.

## Methods

### Rats and treatment groups

The experimental protocols were approved by the University of Saskatchewan Committee on Animal Care Assurance and experiments were conducted according to the Canadian Council on Animal Care Guidelines. Specific pathogen-free, ten-week-old, male Sprague-Dawley rats were procured from Charles River laboratories, Canada. Rats were maintained in the animal care unit and were acclimatized for a period of one week and randomly divided into six groups of five each (Table 1).

**Table 1:** Experimental design

Hours after instillation of	1	6	12	24	36
LPS	n = 5	n = 5	n = 5	n = 5	n = 5
Saline		n = 5			

### Acute lung inflammation

The procedure was performed as described before [5]. Briefly, rats were anesthetized by intraperitoneal administration of xylazine (20 mg/Kg) and ketamine (100 mg/Kg). The trachea was dissected surgically and endotoxin-free saline (Sigma, St. Louis, MO, USA) or *E. coli *LPS diluted in 80 μl of saline (250 μg; serotype 0128:B12; Sigma, St. Louis, MO, USA) was injected in the trachea. Animals were euthanized at 1, 6, 12, 24 and 36 hours (n = 5 per group) post-treatment and were observed during the post-LPS treatment period hourly during the day after application. Although LPS-treated rats appeared to be sluggish, none of them died prior to euthanasia. Control animals (n = 5) were euthanized at 6 hours post saline treatment (Table 1). Only this time point has been chosen for the control treatment because the influence of saline instillation had only very mild effects as compared to LPS.

### Tissue collection and processing

After induction of deep anesthesia the animals were exsanguinated, and the lungs were obtained. Rat lungs were collected for light microscopy without instillation or perfusion with fixatives to avoid any dislocation of leukocytes within the air space or lung vessels. Lung pieces for histology were fixed in 4% paraformaldehyde for 16 hours. Lungs were processed through ascending grades of alcohol and embedded in paraffin. Five μm sections were cut from 6 lung specimens of each rat.

### Immunohistology

Tissue sections were prepared and stained as described before [5]. Briefly, sections were deparaffinized in xylene and rehydrated in descending grades of alcohol followed by treatment with 5% hydrogen peroxide in methanol to quench endogenous peroxidase. Sections were treated with pepsin at room temperature (2 mg/ml in 0.01N hydrochloric acid; Sigma, St. Louis MO, USA) for 45 minutes to unmask the antigens and with 1% bovine serum albumin (Sigma) to block non-specific binding. Sections were incubated with primary antibodies against rat monocyte/macrophage (1:75; ED1, Serotec Inc. NC, USA) or rat MCP-1 (1:300; Torrey Pines Biolabs, Inc. TX, USA), followed by appropriate horseradish peroxidase(HRP)-conjugated secondary antibodies (1:100; Dako cytomation, ON, Canada). The antigen-antibody complex was visualized using a color development kit (Vector laboratories, ON, Canada). Controls consisted of staining without primary antibody or with isotype matched immunoglobulin instead of primary antibody. Proper quenching of endogenous peroxidase was confirmed by omitting both primary and secondary antibodies.

### Tissue evaluation

**Figure 1:**
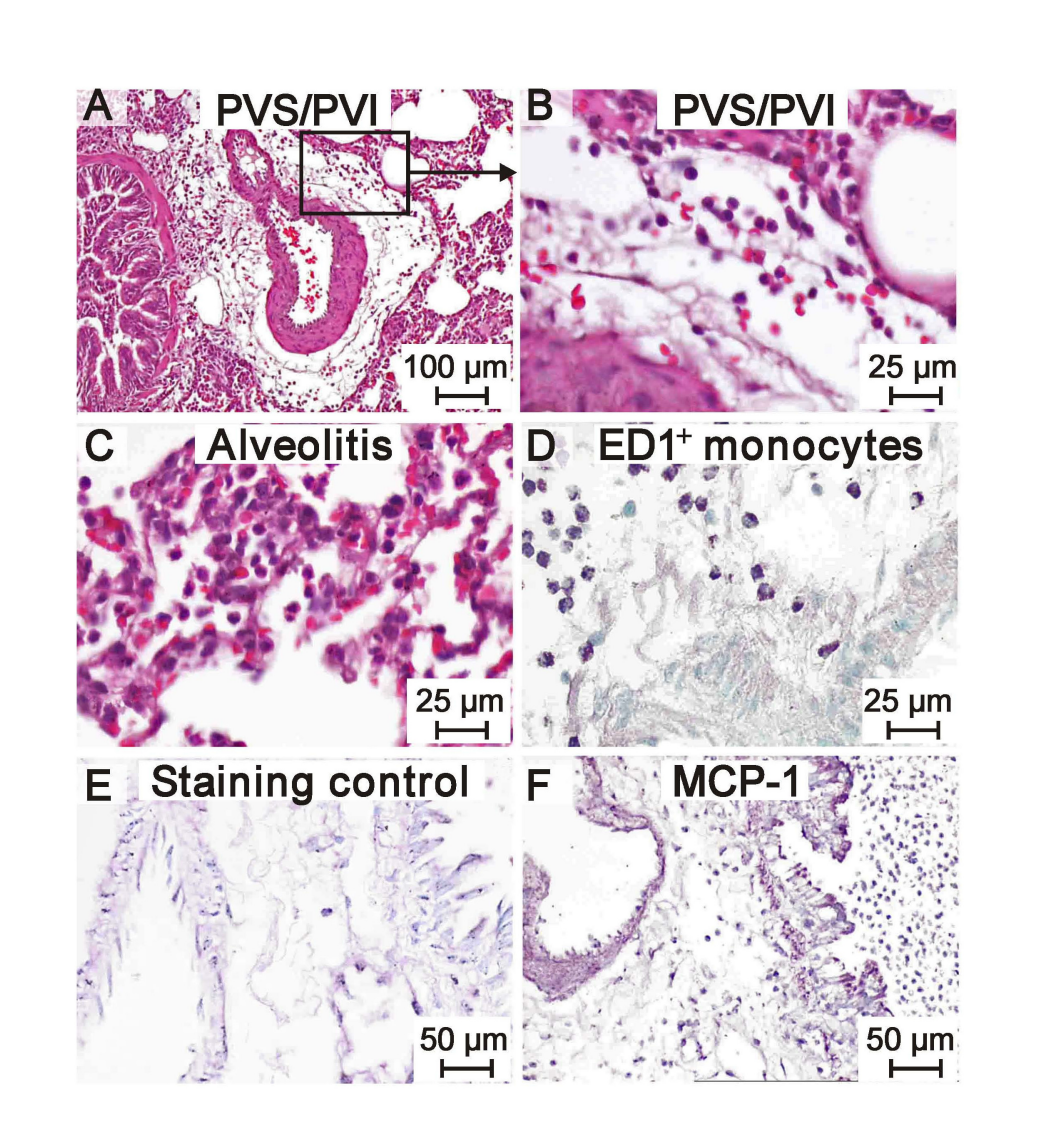
**A-F: H&E histology demonstrating the PVI 12 h after instillation of 25 μg LPS (A, B)**. A massive alveolitis can be seen (C). The leukocytes are mainly ED1 positive monocytes/macrophages (D). MCP-1 expression is demonstrated on the apical epithelium and on the endothelium and most of the leukocytes as well (E control, F MCP-1).

The evaluation was performed by a person blinded to the identity of groups with a microscope using a software assisted determination of edematous area (PVS areas) around pulmonary arteries (PA). We have not evaluated PVS areas around pulmonary veins because the changes there are weaker as compared to pulmonary arteries. Only those PA were included in the analyses, which were fully captured in a cross section and had inner diameter of more than 100 μ. The PVS area was digitally displayed and determined in square microns by the delineation with the cursor. In most of the cases the PVS was clearly separated from the adjacent alveolar tissue as well as the adventitia of the adjacent bronchi and other vessels (Figure 1A). The total number of infiltrating leukocytes was determined using a 200× magnification and the number of neutrophils by using a 400× magnification. Three areas per animal on different lung sections have been evaluated. This semi-quantitive procedure seemed to be adequate because many sections of the same lung revealed similar results as has been checked in single lungs. The cells per area were calculated and statistics performed (MS Office 2003). Mean values (MV) and standard errors (SE) were calculated. Each time point after treatment was compared with the saline treated control group using the non-parametric Mann-Whitney U-test for unmatched pairs and significance was indicated for p < 0.05.

## Results and discussion

Lungs from saline-treated rats showed normal histology and no accumulation of inflammatory cells in alveoli or the PVS. In contrast, lungs from LPS-treated rats displayed a typical accumulation of cells (Fig. 1AB). Single lungs of the LPS-treated rats did not develop edema and perivascular inflammation although they showed alveolitis (Fig. 1C). At time points later than 1 hour after LPS treatment, occasional lymphatic vessels characterized by thin walls and larger diameter were seen in the periphery of PVS and filled with mononuclear and polymorphonuclear cells. Interestingly, aggregates of granulocytes were found within lymphatic vessels after 6 hours of the treatment. The 12 hour groups showed foci of interstitial inflammation which became larger by 24 hours after the LPS treatment and filled most of the section area of peripheral lung tissue after 36 hours. At 12 hours and later, alveolitis and hemorrhages were seen in most of the lungs. The strategy to determine leukocyte kinetics within the PVS was to count in a first step all round cells which are "all leukocytes" in Table 2. These are a) monomorphonuclear cells (MMN) such as monocytes/macrophages and lymphocytes and b) polymorphonuclear cells (PMN) representing the granulocytes. In this model only neutrophil granulocytes could be observed. In a second step the PMNs has been counted separately because only this cell type was changing in numbers very early after the application of LPS. The phenotype of the MMN has not been differentiated in this study because that would be important at later time points in type IV immune reactions. Exemplary the population of monocytes/macrophages in PVS areas has been documentes using the monoclonal antibody ED-1 (Fig. 1D). Low numbers of leukocytes were found in the PVS of the control group and 1 hour after LPS exposition (Table 2). The total number of leukocytes showed a gradual increase in the LPS groups beginning 6 hrs after the challenge reaching significance after 36 hours. In contrast, the neutrophil numbers in the PVS showed an abrupt and significant rise at 6 hours after the intratracheal instillation of LPS, declining to control values at 24 hours. Because MCP-1 is critical for the recruitment of monocytes/macrophages, lung sections were stained with an MCP-1 antibody. Intense expression of MCP-1 (Fig. 1EF) was detected in lung sections from all of the LPS-treated rats especially at time points later than 6 hours in bronchial epithelium, airway and vascular smooth muscles and leukocytes. MCP-1 expression was mild and only in some of the blood vessels including those in the PVS. Lungs from the control rats showed weak staining for MCP-1. To our knowledge, this is the first study to characterize infiltration of neutrophils and monocytes and expression of MCP-1 in PVS of LPS-treated lungs. The study was conducted in a well characterized model of acute lung inflammation induced following intratracheal instillation of *E. coli *LPS. To minimize changes to morphology and introduction of artifacts, the lungs were neither lavaged nor perfused. The histological signs of lung inflammation observed were similar to those reported in various other studies using intratracheal instillation of LPS [4,5,14].

**Table 2:** Density of leukocytes and neutrophils in PVS at different time points after NaCl- or LPS-instillation (cells per mm^2^, mean value ± SEM)

**Group/time**	**All leukocytes**	**Neutrophils**
Saline 6 hours	0.3 ± 0.05	0*
LPS 1 hour	0.3 ± 0.09	0.02 ± 0.02
LPS 6 hours	0.6 ± 0.32	0.2 ± 0.14^+^
LPS 12 hours	0.5 ± 0.23	0.1 ± 0.09
LPS 24 hours	0.6 ± 0.23	0*
LPS 36 hours	0.6 ± 0.09^+^	0*

^+^

significant p < 0.05, *single cells were found

PMN = polymorphonuclear cells/granulocytes (>99% neutrophils)

Our study showed distinct patterns of recruitment of leukocytes into the PVS. The total leukocyte numbers increased slightly 6 hours post-LPS treatment. Significance was calculated only after 36 hours which was due to the moderate increase with high variations and to a small number of animals used in this study. The neutrophils were primarily absent and were increased rapidly after 1 and 6 hours and disappeared again after 24 hours. Compared to the alveolar recruitment leukocytes came slow but neutrophil migration into the PVS was as quick as into the alveoli [5,14,15]. The slight increase of monocytes in the PVS the LPS-challenge was in contrast to our recent data showing clear increases of monocytes within the alveoli already 3 hours after an LPS-challenge [14] indicating the PVS as a compartment which is functional distinct from the alveolar space.

The mechanisms and route of recruitment of inflammatory cells into PVS remain largely unknown. Recently, we reported that there are strain-dependent differences in inflammatory cell recruitment into PVS in acute and chronic airway inflammation [16]. Although we showed expression of Vascular Adhesion Protein (VAP-1) in pulmonary arteries in a mouse model of airway inflammation, we are aware of a significant structural barrier afforded by their thick wall [17]. Therefore, we believe that entry and existence of inflammatory cells into PVS possibly occur through capillaries and lymph vessels present in the PVS. However, other authors believe that cells exit the blood stream and pass immediately into the PVS [13]. This is supported through our direct observations of neutrophils in the lumen of microvessels in the PVS. One of the critical requirements for inflammatory cell recruitment is expression of chemoattractants [15]. A classical chemoattractant for monocytes/macrophages is MCP-1. Our data show expression of MCP-1 in recruited cells in PVS along with airway epithelium and vascular endothelium. Immuno-histological localization of chemokines such as MCP-1 is difficult and does not provide direct information on their functions. The intense expression of MCP-1 observed in PVS in lungs of LPS-treated rats may indicate its role in promoting monocyte/macrophage entry into PVS.

## Conclusion

We conclude that PVS may be a unique anatomical and functional site for the migration of inflammatory cells in acute lung inflammation. Therefore, PVS may contribute to immune responses in lung inflammation provoked through various stimuli. We still need to answer intriguing questions such as the route and mechanisms of migration of inflammatory cells into PVS.

## Competing interests

The authors declare that they have no competing interests.

## Authors' contributions

KSJ performed the animal experiments and was involved in the morphological evaluation and helped to draft the manuscript. TT performed the analysis of the lung sections and drafted the manuscript. RP was involved in coordination of the study and helped to draft the manuscript. BS conceived of the study, participated in its design and helped to draft the manuscript. All authors read and approved the final manuscript.
